# Harvard Medical School Department of Global Health and Social Medicine COVID-19 seminar series: COVID and surgical, anesthetic and obstetric care

**DOI:** 10.1186/s12919-021-00218-3

**Published:** 2021-09-23

**Authors:** Elizabeth Miranda, John G. Meara, Alaska Pendleton, Alexander W. Peters, Vatshalan Santhirapala, Nabeel Ashraf, Nivaldo Alonso, Sadoscar Hakizimana, Abebe Bekele, Kee B. Park, Paul Farmer

**Affiliations:** 1 Program in Global Surgery and Social Change, Harvard Medical School, 641 Huntington Ave, Boston, MA 02215 USA; 2 Division of Vascular Surgery, University of Southern California, Los Angeles, CA USA; 3 Department of Plastic and Oral Surgery, Boston Children’s Hospital, Boston, MA USA; 4 New York–Presbyterian Weill Cornell Medical Center, New York, NY USA; 5 School of Medicine, Imperial College London, London, UK; 6 Indus Health Network, Karachi, Pakistan; 7 Department of Plastic Surgery, Faculdade de Medicina da Universidade de São Paulo, São Paulo, Brazil; 8 Partners In Health – Rwanda, Kigali, Rwanda; 9 University of Global Health Equity, Butaro, Rwanda; 10 Department of Global Health and Social Medicine, Harvard Medical School, Boston, MA USA

**Keywords:** COVID-19, Surgery, Surgical healthcare, Global health security

## Abstract

On May 21, 2020, the Harvard Program in Global Surgery and Social Change (PGSSC) hosted a webinar as part of the Harvard Medical School Department of Global Health and Social Medicine’s COVID-19 webinar series. The goal of PGSSC’s virtual webinar was to share the experiences of surgical, anesthesia, and obstetric (SAO) providers on the frontlines of the COVID pandemic, from both high-income countries (HICs), such as the United States and the United Kingdom, as well as low- and middle-income countries (LMICs). Providers shared not only their experiences delivering SAO care during this global pandemic, but also solutions and innovations they and their colleagues developed to address these new challenges. Additionally, the seminar explored the relationship between surgery and health system strengthening and pandemic preparedness, and outlined the way forward, including a roadmap for prioritization and investment in surgical system strengthening. Throughout the discussion, other themes emerged as well, such as the definition of elective surgery and its implications during a persistent global pandemic, the safe and ethical reintroduction of surgical services, and the social inequities exposed by the stress placed on health systems by COVID-19. These proceedings document the perspectives shared by participants through their invited lectures as well as through the panel discussion at the end of the seminar.

## The timeline of COVID-19 and its relationship to surgery – John G. Meara, MD

In late December, Wuhan, China reported a case series of pneumonia that turned out to be caused by the coronavirus, COVID-19. By the end of January, the WHO declared a public health emergency, and 6 weeks later, the WHO declared COVID-19 a pandemic. Now coronavirus is essentially ubiquitous. It is everywhere. It has traveled around the globe. And we have all lived through this.

There have been some very granular, specific topics in the surgical literature. Articles about emergency procedures, changes, and protocols that came up very rapidly, what has happened to the SAO workforce, how to conserve personal protective equipment (PPE) in a surgical environment, and so on. This is one end of the spectrum, the very granular perspective from the front lines. The other end of the spectrum, though, is more philosophical. What is the link between surgical care and pandemic preparedness, at a national level or a global level?

Dr. Meara argued that we can go back about 400 years to frame this. The Treaty of Munster and the Peace of Westphalia, created in 1648, formed our modern conception of sovereignty. This was the end of the 30 Years War and the 80 Years War, and this is where the concept of sovereign autonomy came from. This meant that sovereign countries had no right to invade other countries, but it also had a corollary which led sovereignties to feel like they had no responsibility to other countries.

About 400 years later, an article by Richard Haass in *Foreign Affairs* turned this concept of sovereign autonomy upside down. It argued that countries should have a feeling of sovereign obligation, that we have a responsibility to other countries into the world [1]. And this article was not necessarily a health article – while a paragraph or two focused on health, it also discussed a number of different issues with sovereignty. An online discussion with Richard Haass about this message led to an article that Dr. Meara published with Brian Till [2]. They took his concept of sovereign obligation a little further, particularly in health care, with two major premises. The first is that global health security is promoted by holistic, strong, stable health systems everywhere. In other words, health deserts anywhere are a threat to people everywhere. The second major point was that health equity and social justice are really best promoted through this concept of sovereign obligation, not autonomy or fierce nationalism. And you wonder, is that still an issue today?

Yes, it is still alive and well, this sense of fierce nationalism, as evidenced through a recent MSNBC article entitled, “A wave of vaccine nationalism hinders global efforts to halt coronavirus.” [3] This is certainly still an issue.

So in terms of global health security and surgery, what have we learned from COVID? Dr. Meara left the speakers with three thoughts to consider:

- What is the role of transnational cooperation and transparency?
- What is the role of surgical capacity and surgical workforce as a reservoir of critical care capacity for pandemic response?
- What have we learned about our prior concepts of health system strength and structure?

Dr. Meara brought up these points because prior to coronavirus, the United States had the highest ranking for global health security. And we all know how the United States and in particular the East Coast, fared, despite the fact that the US system spends the most on health care. Consider that New York City has more staff, stuff, and space than maybe any city in the world, and yet they did not fare well. That might speak to the United States approach to systems and the fact that we may have concentrated on hospital systems, but not community systems, which are equally important to health. And then you look at another system, the United Kingdom. The UK has a national health service, but they also did not fare well with coronavirus. So maybe our prior view and structure for national systems was not well-suited to dealing with this.

### Dr. Paul Farmer’s reflections

By January 12th, Chinese authorities had published the genetic sequence of the virus, a true reflection of the urgency and speed of the early response to the outbreak, as well as a reminder of what tools we do have, if we can marshal them. When we have a vaccine, it will be in part because of the speed with which that genetic sequence was published.

The idea of sovereignty and the nation-state have a history. One of the ironies about what happened after the Treaty of Westphalia to our globe is that the people living on it were really subjects, not citizens. And within a century of this groundbreaking treaty, the world would be carved into colonies. Just to give an example, the place where the largest number of colonial subjects lived, for at least a couple of centuries, was India. But this spread of colonial rule in the late 19th and twentieth century had enormous implications for the discussion we are having. It would take some time, with the continent of Africa the last to fall. And that was largely a late-nineteenth century, twentieth century phenomenon. One of the deeper questions that we can ask is, what does it mean when sovereignty is stripped away, not just in terms of the nation state, but in terms of people who are not citizens but subjects?

There has never been a great leveler like COVID-19. It is hard to imagine another pathogen that.

could come in and say, well, everybody’s at risk, because nobody is immune, though it is essential to take into account the gravity of the social disparities that we see in both infection and case fatality rates. We did hear those kind of comments early on, but they were stilled by the gravity of the social diparities that we see in both infection and case fatality rates. And we should be returning to that as well.

## The Massachusetts General Hospital COVID-19 Bundled Response for Access (COBRA) team – Alaska Pendleton, MD

From the time that the COVID impacted the Massachusetts health care system in mid March, Dr. Pendleton was involved in clinical care within two primary contexts: first, working in a COVID ICU, and second, working as part of an acute procedures team known as the COVID-19 Bundled Response for Access team, or the COBRA team.

When the COVID surge first hit Boston, Dr. Pendleton was working in a burn unit, which was converted to a COVID ICU over a few hours. Within the course of one shift, burn patients were wheeled out on stretchers as the ICU COVID patients were brought in on travel ventilators, many of whom needed to stay on these portable ventilators given limited ventilator supply. This was illustrative of the fact that over the course of the COVID surge, Massachusetts General Hospital (MGH) had over 12 fully supplied COVID ICUs. Many of these ICUs were previously neuro or cardiac step-down units or, in this case, a burn unit.

At the end of April, MGH had over 165 COVID ICU patients alone, not to mention floor or emergency department patients, creating a need for skilled physicians to place challenging access for these patients. As many of the physicians in the audience may have experienced or realized from their working with COVID patients to date, these patients pose a variety of challenges for vascular access. They are hypercoagulable - if you ultrasound their upper extremities, you often demonstrate significant clot burden, limiting access sites. Additionally, obesity is a risk factor for severe COVID illness, and body habitus poses another additional challenge to line placement. Finally, as the average ICU stay was on the order of 14 days at MGH, nearly all these patients had prior lines that had thrombosed or other lines that were still in usage, again limiting access site availability.

To address this need, the Massachusetts General Hospital surgery department created a multidisciplinary team of anesthesia, surgery, and interventional radiology providers. This team was put together largely due to the outstanding effort of Dr. Kat Albutt, who was a former PGSSC fellow, as well as Dr. Casey Luckhurst, who are both current residents at MGH. The goal of the team was to provide safe, streamlined, and bundled vascular and enteral access. This effort aimed to increase patient safety by having a trained team perform these procedures and to protect ICU providers by limiting COVID exposure and preserving our PPE. This was a 24/7 service with four full-time day residents and two night residents with a supervising attending at the peak of the COVID surge in April and early May.

**Fig. 1:**
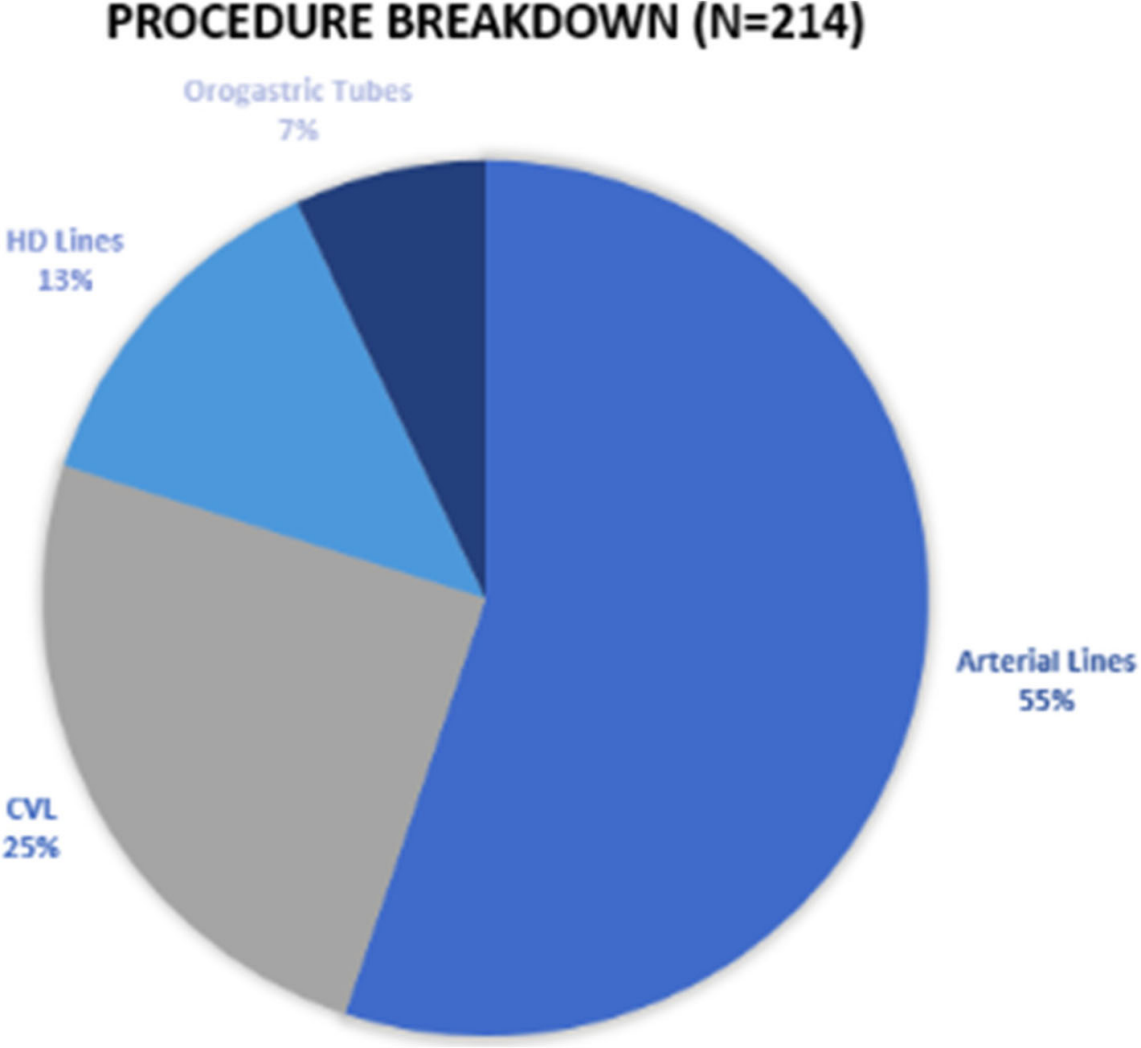
Procedure breakdown

Figure 1 indicates early on the procedure breakdown, with arterial lines being the most common procedure performed. This is not surprising given the recurrent thrombosis arterial lines suffered, often requiring femoral or sheath access. This is followed by central lines and dialysis access.

The COBRA team represented a combined effort between surgery, anesthesia, and interventional radiology to provide an essential service for medical services stretched extremely thin during the surge. On an institutional scale, the streamlined team manifested how surgical workforce could be effectively leveraged to provide support in a pandemic situation. And this provided a reservoir of critical care capacity as previously referenced by Dr. Meara. The team received so much positive feedback that there was interest in continuing the service beyond the initial COVID surge.

### Two months later

Massachusetts reached a COVID-19 surge peak at the end of April and by August 2020 had over 118,000 cases of COVID and over 8600 reported COVID deaths [4]. Whereas at the surge peak MGH had over 160 patients with COVID requiring ICU-level care, as of the end of July there were less than 5 patients in a MGH ICU for treatment of COVID. The COBRA team was tapered and disbanded in June given decreasing need for access procedures. There remains ongoing discussions with program directors for how the training model may be integrated into medical and surgical residency programs in the future.

## COVID-19 and surgery in New York City – Alexander W. Peters, MD

The New York-Presbyterian Hospital system is an 11-hospital system which includes Columbia, Cornell, and a number of hospitals in the New York City area. New York was particularly hard hit by COVID-19, with over approximately 222,000 cases, approximately 56,000 hospitalizations, and approximately 23,000 deaths by August 1.

At the peak in April, the hospital system, which normally has a capacity of 2100 beds, had approximately 2600 COVID-19 positive inpatients admitted. This was in addition to any other COVID-19 negative patients admitted for care during this time. At the same time, there were approximately 760 COVID patients in ICUs requiring ventilator supports, nearly double our 420 ICU bed baseline capacity. To meet this surging demand and expand hospital bed and ICU capacity, the hospital system developed several rapid solutions across the hospital system.

One of the efforts that Dr. Peters worked on was the conversion of many of the operating rooms (ORs) into ICUs. To do this, a framework was developed for an ICU conversion process that applied to both recovery rooms, and ORs. The questions asked were: 1) if we can do this at a given hospital; 2) in which ORs and recovery rooms is it possible; 3) how many beds can fit in each space; 4) what equipment will be needed; 5) what will the care team needs be; and finally, 6) what patients should be assigned to these new beds? The hope was always that the answers to the previous questions, in terms of capacity, would exceed the demand of the last question.

Several OR clusters for were selected conversion. The OR sterile core was emptied out and this spaced restocked with all the supplies needed for ICU care. Normally this is full of sutures, staplers, and other OR equipment that is not needed for ICU patient care. Then it was assessed how many patients could be fitted into each OR. The team unfortunately had to forego isolating individual patients and instead needed to cohort COVID positive patients together to make more space. Dr. Peters explained how it was marked out on the floor how many beds and ventilators and other equipment we could fit as this was planned and then planned which ORs could be made into negative pressure rooms. The facilities team was able to change the ventilation in ORs which normally provides positive pressure and install HEPA filters to clean the air. Specialized filters were placed over air ducts, turning all of our ORs into negative pressure environments to try and reduce the infectious spread.

The hospital did not have additional ventilator for the ORs. Instead, the team converted all of the anesthesia machines during this process and used them as ventilators. Similarly, the team used mayo stands, the OR lights, and a number of other things to our advantage to equip our new ICUs. Once it was determined if they had all the equipment to do this and how many beds could be allocated for ICU space, they moved on to determine how to staff these new OR ICUs, as they came to be known. CRNAs played a huge role in this because most providers do not know how to use anesthesia machines as ventilators. CRNAs are very familiar with the anesthesia machine function and therefore served as respiratory therapist in the ICU.

This OR to ICU conversion was a very rapid process. Dr. Peters explained that they were racing to get ICU beds open, and on the fifth day after initiating this project, they opened the first OR ICU. They opened a total of 34 OR ICU beds, and expanded the same process across several of the recovery rooms as well, opening up 60 new ICU beds at Cornell alone and replicating this process at Columbia and a number of other hospitals across our system.

**Fig. 2:**
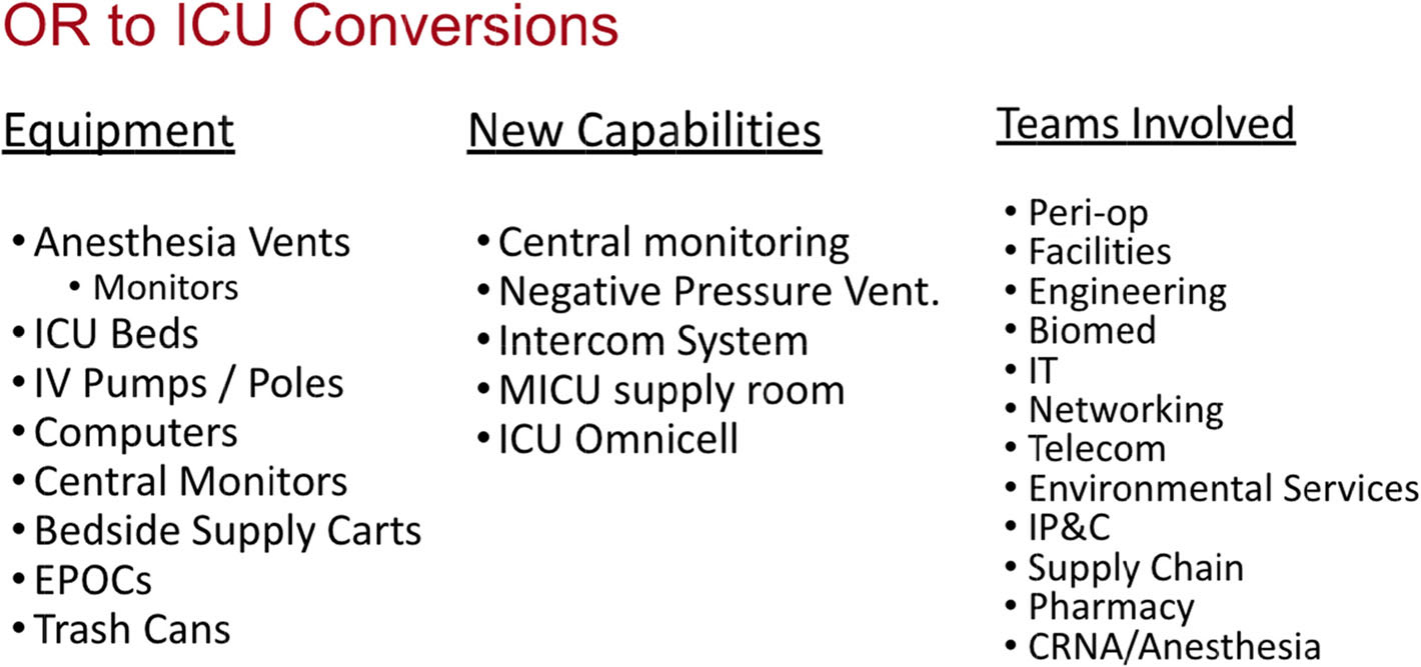
Equipment, capabilities, and teams for OR to ICU conversion

Figure 2 outlines the new equipment, capabilities and teams needed to implement the OR to ICU conversion. It highlights some of the new capabilities that we built into these ORs, including the negative pressure system, an intercom system, and central monitoring systems that they don’t normally have, as well as a hugely cooperative, multidisciplinary effort needed to make this happen. It was not any single team that drove them forward, but a real group effort.

Dr. Peters outlined three takeaways from this rapid process: First, this is possible in ORs. You can surge ICU capacity across many different places using this framework. Second, staffing was absolutely critical and the rate limiting step. Staffing shortages initially prevented the institution from opening up some the OR ICUs when they were ready. Finally, it takes a hugely multi-disciplinary team to make this happen. But it is possible, and it helped to meet the surge in New York.

## COVID-19 response and outcomes in London – Vatshalan Santhirapala, MBBS

St. Thomas is fortunate to be located just across the road from House of Parliament and Big Ben and adjacent to the River Thames. It is as central as you can get for hospitals, and it also had the highest burden of COVID patients in the whole of the UK, and had 1500 PCR positive patients, with over 300 ICU admissions and a surge capacity of 188 critical care beds from a normal baseline of 50. Following a request by the NHS England, they expanded up to 300 beds, but the surge diminished quite quickly, possibly due to the lock down.

Dr. Santhirapala explained that St. Thomas had a high number of ECMO beds, a total of 25. When ventilation fails, ECMO was really the only option. They had the largest ECMO center in the whole of Europe, followed by Karolinska Institute, and St. Thomas had the highest COVID bed status for ECMO for a single institution.

**Fig. 3:**
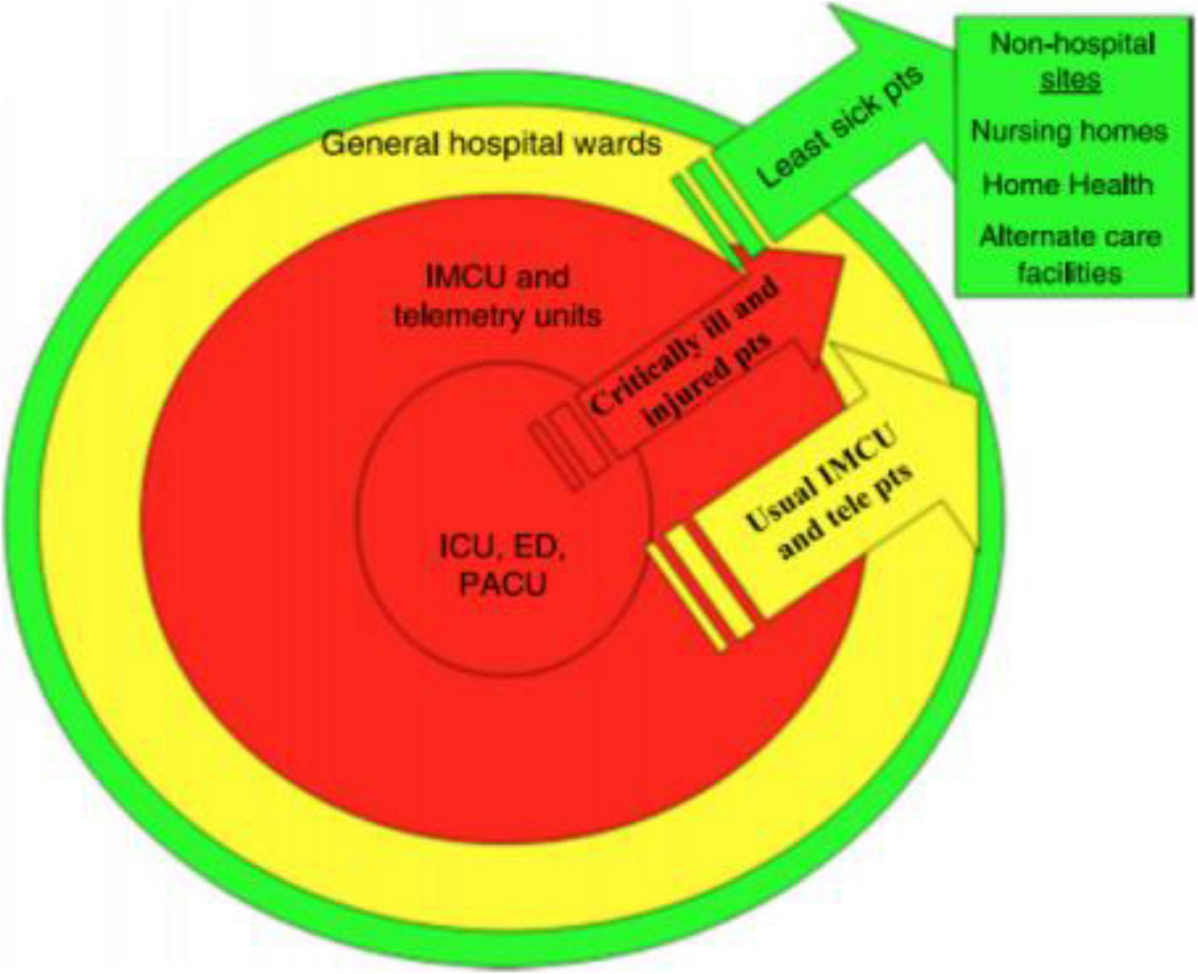
Anesthesia & Critical Care COVID-19 surge planning

Resources alone will not allow one to meet the care demands created by a pandemic – strategy is essential as well. The framework for their strategic thinking can be seen in Fig. 3**.** The red sections in the circle represent critical care bed needs and ED bed needs, needs to be enhanced within the hospital network. In order to do that, the team needed to stop the general ward patients, represented by the green sections of the diagram, from coming into hospital. They did that by canceling all elective surgery and all procedural interventions by the third week of March. These, obviously, have secondary health care outcomes, the impact of which is still unclear. Urgent care surgery did occur on an off-site location.

The strategic meetings occurred in a medical school boardroom, where a chalkboard was used to write up the staff, supply, systems, et cetera. That’s how they oriented the strategic meetings every day and went through supplies of PPE, staffing ratios, etc. on a daily basis.

**Fig. 4:**
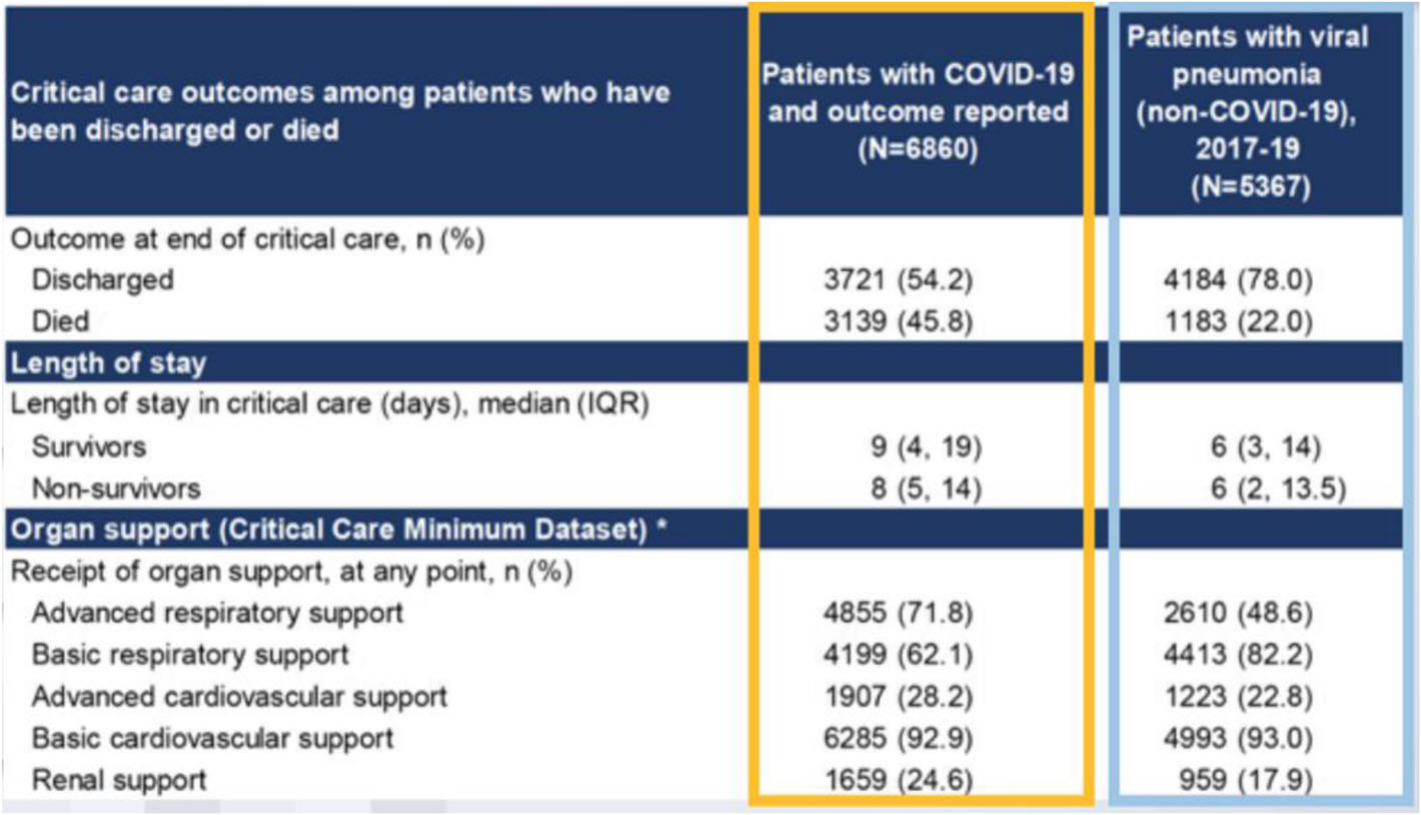
COVID-19 Critical Care Outcomes

Figure 4 shows some of the outcomes that were seen in the UK, which has a very robust audit system for critical care outcomes. The yellow box looks at COVID-reported outcomes, and the blue box looks at non-COVID viral pneumonia. The critical care mortality from the UK population for COVID-19 was 46%, versus the 22% influenza. There was a high burden of patients that need advanced respiratory support, and what they didn’t quite expect is that a high proportion of patients also needed renal replacement therapy, with approximately one in four patients needing dialysis of some sort. St. Thomas Hospital had to adopt novel dialysis mechanisms, such as using sustained low-efficiency dialysis, or SLED, instead of continuous hemodialysis. They also had to stop producing their own dialysis fluid, because stocks were running quite short.

The last thing Dr. Santhirapala described was the ethnic disparity seen in the UK. St. Thomas was already looking at this under the microscope. The Asian cohort of patients had the highest mortality in the UK, at approximately 52.5%, that was really only half the story. If you compare the outcomes to what you get as an influenza cohort, you see that among African, Caribbean, and mixed ethnicities, there was a fourfold increase in mortality than you would expect from normal viral pneumonias. There may be some genetic component, but undoubtedly there are underlying socio-economic disparity in the social infrastructure in the UK. COVID-19 exposes this social disparity that they were seeing.

### Dr. Paul Farmer’s reflections

There is still reference to the special relationship between the UK and the United States. Well, it is clear that we still have that special relationship in terms of the ways in which our social inequalities lead to radically differing risk of infection for people of color, however they are described in the local parlance, and also for increased risk of mortality. There is a lot more to learn about why mortality is lower in some places than others, but it is a very strikingly similar picture to what we’re seeing the in United States. It highlights the similarities between social infrastructures in the UK and the United States, ways in which our social inequalities lead to radically differing risk of infection for people of color and increased risk of mortality. And it is not the kind of special relationship we want to share.

## COVID & ICU care in Pakistan – Nabeel Ashraf, MBBS

Dr. Ashraf explained that he had been working since the end of March on trying to improve the capacity of Pakistan’s health system in providing care to the COVID patients and dealing with the pandemic. Pakistan is a country of 200 million people, and Dr. Ashraf belongs to the country’s largest city, Karachi, which has a population of 16 million.

Pakistan’s economic challenges and fragmented health system left it unprepared for this pandemic. However, when push came to shove, Pakistan took many commendable initiatives from the federal level as well as different provincial governments took their own measures. These included travel bans, lock downs, and physical distancing. Through district health offices, they initiated screening, contact tracing, community quarantines and isolations. Both private as well as public sector labs were involved to increase testing capacity. Governments began working on creating isolation facilities as well as the critical care units for COVID patients.

With 3 months since the first case was registered, the count of cases was 42,000 at the time of the webinar. But as the lockdown was being eased for economic reasons and people were interacting, there was a fear that the number of cases will increase and the death count of around 900, which is not a very high death rate at 2.1%, may also increase.

One of the problems is that Pakistan’s ICU capacity is not optimal. It is reported that Karachi, which.

is the biggest city of Pakistan, has only 480 critical care beds, out of which only 40 to 50% of the beds have ventilators [5]. Moreover, only 7 to 8% of these beds are manned by dedicated consultant specialists [5]. Pakistan is therefore heavily dependent on nurses for the management of ventilated patients. In case of an increase in complicated cases, Pakistan might have a lot of deaths. Dr. Ashraf and his colleagues hoped that the strain is kind and does not lead to complicated cases, so that we do not need a lot of ventilators.

In March, Dr. Ashraf worked with a team of volunteers, army personnel, and government doctors to create an isolation facility. They converted the largest convention center in Karachi to make a 1200-bed hospital. Within a short period of 2 weeks of teamwork, they were able to operationalize the isolation center; the army led the infrastructure changes while the government helped in mobilizing the required finances and human resources, and the young medical graduates like Dr. Ashraf along with senior doctors developed standard operating procedures.

### Two months later

Following the ease in the lockdown and the religious festive occasion of Eid ul Fitr on the 22nd of May, a steep increase was seen in the number of COVID cases. The count of cases and deaths increased from 42,000 cases and 900 deaths to 249,397 cases and 5999 deaths as of August 3rd 2020 [6]. The highest number of daily new cases, 6825 cases, was seen on 13th June 2020.

However, with dedicated awareness campaigns, smart lockdowns, and compliance to standard operating procedures by the people, Pakistan saw a downward trend of cases. The national COVID-19 dashboard website (http://covid.gov.pk/stats/pakistan) set up by the Government of Pakistan played an important role in informing the population about the severity of the pandemic. With easy to understand charts, the dashboard kept people updated about the country’s status. Currently, the curve is flattening; cases had steadily decreased to less than 500 cases with only 330 new cases recorded on the 2nd of August [6].

With the sudden increase in cases in the start of June, Dr. Ashraf’s team at the Field Isolation Center decided to develop High Dependency Unit beds for oxygen dependent COVID patients. A 140-bed unit was created in one of the convention halls. Infrastructural changes, equipment, supplies, human resource and training requirements were listed, and standard operating procedures were developed for providing services to the critical COVID patients who were not able to receive care at the already saturated hospitals of the city. The HDU at the Field Isolation Center was operationalized on the 23rd June 2020.

Now that the curve was flattening, life was returning to normal. However, there was emphasis from the government to make sure basic prevention SOPs are followed by the public. Due to cases of reinfection, there was also awareness about a possible resurgence of COVID. The COVID wards established at hospitals across the country will continue to exist to provide care to patients with COVID. Field hospitals planned to stay for another month in anticipation of a possible second peak. COVID tests are being done before surgical procedures and travelling while all types of healthcare is being provided with the appropriate personal protective equipment. While there was focus on testing possible vaccines, there was an understanding among the public and healthcare professionals that COVID is here to stay and the appropriate precautions have become part of routine life and healthcare practice.

## COVID, surgery, and surgical training in Brazil – Nivaldo Alonso, MD

The plastic surgery department is located in the central building of the University of São Paolo, with 1000 beds available. Now, that whole building is reserved for COVID patients, and the plastic surgery departments are now working in the orthopedic and cardiac building. They have more than 250 patients hospitalized in ICU and over 300 other patients in the other beds in this central building.

No other procedures were being done in this central building, and very few elective procedures had been done, only the ones that are really necessary. Very few patients with COVID had been operated on - in the week prior to the webinar, only three patients needed some kind of plastic surgery procedures to be done. Dr. Alonso’s last surgery was in the third week of March.

They also had the emergency department open. The number of trauma cases and other cases in emergency were drastically reduced. But even if there was a decreased trauma burden, likely due to decreased traffic or maybe even decreased gunshot wounds, Dr. Alonso explained that it was very hard for surgeons to stay at home. They also have a medical school and were teaching classes for the students and giving lectures for residents.

A lock down was planned until end of May, and then they would decide what was going to happen next. Dr. Alonso reiterated the difficulty of the situation and how difficult it is for surgeons to be unable to enter the operating theaters. He also emphasized that São Paolo is a very special city in the country, and that maybe in some other small cities, the situation could be different. He also works in Bauru, a small city, and they still had some elective procedures, but their number of patients with COVID was much less.

### Two months later

Dr. Alonso explained that 2 months later, they were facing a totally different situation in São Paulo. All the cities in the countryside were hard-hit with COVID-19 and in the capital the situation was stable, and things were almost back to normal in private specialty clinics. Also opposite to the situation described in May, Dr. Alonso’s Plastic Surgery Department was very busy with COVID-19 patients due to high incidence of pressure sores and others lesions related to long hospital stay for the patients. Elective procedures with very strict rules were now allowed in private Hospitals. The University Hospital was now being prepared to restart consultations and surgeries in August.

Five months later from the beginning of the pandemic, 98% of all Brazilian cities had been affected (5.442) with more than 93.000 deaths. Surgeries in Dr. Alonso’s hospital had decreased more than 50% and rate of mortality had increased over 20%. Surgeons at the Emergency Unit made a new classification for surgeries, not just elective and emergent, but also elective urgencies, such as bowel obstruction for cancer, and essential elective, for time dependent surgeries.

Dr. Alonso said that this pandemic is a big challenge for the Brazilian SUS - Universal Health System. Social inequality and economic differences among states brought up difficulties in accessing good health care for a large part of the population. Even if their health system is considered a good model, it still needs new concepts and strategies to deal with workforce and facility challenges.

### Dr. Paul Farmer’s reflections

As we talk about reopening a city like São Paolo or Boston, or any others, you would think that we could get restarted quickly on surgical cases. This is especially the case since surgeons and others in the ER – the surgical, obstetric, and anesthesia supportive staff – are familiar with PPE in a way that is likely not the case for most other sub-specialties, who are not as familiar with protecting themselves from spread from these sorts of pathogens. Given this, surgeons have a special obligation, just like nations do, to share their knowledge of how to protect themselves from pathogens like this.

## The effect of COVID on surgical and obstetric care delivery in rural Rwanda – Sadoscar Hakizimana, MD MMed

At the time of this webinar, there were a total 257 cases in Rwanda, with 123 active cases, and there were done a total of 44,245 tests. There had been no deaths to date. The role of the district hospital, like Kirehe, was to screen, isolate, test, and refer positive patients to a national treatment center. A number of measures had been put in place by Kirehe District Hospital and Partners In Health to protect patients and staff.

Dr. Hakizimana explained that the pandemic had changed his surgical and obstetric practice by affecting his ability to care for patients. For example, post-operative hospital stays were increased because of transport difficulties for patients to get home. There was a delay in access to care because of the lockdown, and the limited access to transportation means difficulties to get health facilities. The transport problem was observed in the beginning of lockdown but was resolved through a good communication and collaboration between patients, administrative authorities, and the hospital and health centers.

Outreach clinics, which provide antenatal care had been closed, and mentoring and supervision activities were on hold. There was an increased number of emergency c-sections, which negatively affects the safety of childbirth. Furthermore, hospital resources, such as human resources, were diverted away from surgical and obstetric care delivery to respond to COVID-19. Elective surgeries had also been delayed, which was anticipated to be a burden for the coming months. Dr. Hakizimana anticipated an increase surgical disease burden, which would need additional resources such as staff, space, and equipment. And even though Rwanda had spent a great deal of time preparing, Dr. Hakizimana feared that there were still going to be poor outcomes because of this decrease in prenatal care and lack of elective procedures.

But he explained the lesson they learned from the COVID pandemic – nobody is safe until everybody is safe. The world is like a plane. If the plane crashes, everybody crashes, no matter where you are – in economy or business class – or who you are, even if you are the pilot.

### Two months later

As of August 3, the total number of confirmed cases in Rwanda had risen to 2099 with only 5 total deaths. The number of testing sites had increased to seven total, with three located in the capital of Kigali and two more each in the Eastern and Western provinces, where hotspots had emerged. Additionally, the number of treatment centers had been expanded to 18, with centers in each province and the eventual goal to have one treatment center in each district.

In Kirehe district, the local government is working with Partners in Health to train employees in infection prevention & control (IPC) measures, with a total of 57 staff members undergoing the training, including health and sanitation officers from different sectors, community environmental health officers, and youth volunteers. Furthermore, IPC materials, such as PPE and cleaning materials, were provided to quarantine sites and accommodation centers, along with mentorship on IPC measures.

Additional materials had been requested for cesarean sections and other obstetrics and gynecology cases, and a temporary delivery room had been set up in Kirehe District Hospital for COVID-suspected emergency obstetric cases. Regular decontamination of rooms and cars that accommodated suspected COVID patients had been performed.

Well-defined roles and responsibilities have been key to Rwanda’s COVID-19 response. Community health workers have undergone education on home-based isolation, care, and IPC measures. The Ministry of Local Government is responsible for reinforcement of isolation and quarantine measures, monitoring of compliance to set rules, community policing, assessing of food security, and distribution of food and water to those in need. The Ministry of Health in conjunction with the Rwanda Biomedical Center developed standard operating procedures and guidelines, defined referral procedures and financial mechanisms, monitored implementation & review of these guidelines, and mobilized resources to support implementation of these policies. Finally, development partners have provided technical and financial support for the implementation of the home-based isolation and care guidelines.

## COVID-19 and surgical care in Africa – Abebe Bekele, MD FCS FACS

Dr. Bekele began with an overview of the COVID-19 situation on the African continent at the time of the meeting. As of late May 2020, the total number of COVID patients had reached close to 100,000, with close to 3000 deaths, yielding a case fatality rate between 3 and 3.5% [7]. The numbers seemed lower as compared to when the first case was reported about 7 weeks prior. Dr. Bekele posed the question of whether this was due to limited testing capacity in the continent, or because countries were testing a limited number of suspected cases. Was Africa suffering from the milder form of the disease that is considered non severe, and patients don’t even report to hospitals? Whatever the case may be, the numbers at that point were still low. But the numbers were definitely increasing, and from the cases reported every day across the region, most patients did not have travel history. There was clear evidence that there is community transmission, and it was, in fact, out of control in some countries. Additionally, some countries were not reporting case numbers at all. So, one might even question, is modeling applicable in this continent or in this setting?

Access to both elective and emergency surgery was compromised. At the time of this webinar, 15 countries across the East Central and Southern African region had completely cancelled elective surgery. Dr. Bekele then discussed how the definition of “elective” probably has shifted significantly over the past few months. The classical definition was not valid anymore. How we used to define “elective” was non-life threatening, and an added component of time has been previously used to specify “urgent” and “emergent.” That was what we used when the pandemic hit, when we did not know how to behave as professionals and as care providers. After knowing that the disease would be with us for quite some time, we had to redefine what we mean by “elective.” Cancer cases, pediatric cases, cases that deteriorate into emergencies and become life-threatening within a short period of time, should not be considered as classical “electives”. Studies had already shown that more than 50% of the so-called elective cases changed into emergencies in a short time [8]. At the time of the webinar, only emergencies and urgent cases were being operated on, and in two or three countries, pediatric and cancer cases were also given priority. Hence, operating waiting lists were quickly getting out of control, with some waiting lists ranging from 8 months to 1 year.

A recent publication showed the number of cases that were canceled as a result of the COVID over the past 12 weeks [8]. Close to 28.4 million surgeries worldwide were being canceled, and 2.3 million cancer surgeries were either canceled or delayed [8]. This is especially true in the developed world, where, for example, cancer patients were being diagnosed at a late stage to begin with. Add cancellations, and we can easily understand what that means. So Dr. Bekele proposed that it would be, in fact, better not to talk about how we handle emergencies versus “electives” in this era, or what electives mean, but rather how to reintroduce surgical services in a safe and equitable manner. Dr. Bekele and his colleagues had recently published a guideline in the *Annals of Surgery* about COVID preparedness and surgical care in Africa, how surgeons, anesthetist, and surgical care providers should prepare their services and theaters, about testing of surgical patients, about PPEs and how to gradually go back to normal [9].

Even if emergency cases were being operated on, some countries had instituted a very strict lockdown, which means travel between the rural setting and the hospitals was compromised. So patients could not get transportation to come to hospitals for urgent and emergency care. By travel, Dr. Bekele also meant travel of health workers. In Africa, it is unusual for health providers, including nurses, lab technicians, and doctors, to drive or to have a car. Sadly, there were reports of discrimination against health care providers while using public transports. Some were even kicked out of houses they were renting. Most use public transport, which were completely stopped. So it was not unusual to see health professionals unable to even go to work because of the lockdown. A strict lockdown also meant many – in fact, the majority – of Africans who depend on daily wages do not have access to health care. So the restrictions by themselves compromised the access to surgical care.

Regarding COVID and the surgical workforce, to begin with, Dr. Bekele explained that the region suffers from extremely compromised SAO workforce density. And at the time of the webinar, the SAO workforce was made ready for the coming catastrophe. Anesthetists and surgeons were being prepared to serve as intensivists. Junior professionals such as residents and interns would be in the emergency rooms, and the entire workforce, then – the very small, limited workforce – would be diverted towards COVID care. And most hospitals, and not only hospitals but also hotels, private apartments, and big meeting halls, were being readied and utilized for patient care.

And it was not only SAO workforce, but also SAO resources, that were being diverted to COVID. Most countries were seeing serious shortages of blood, as blood donation was severely compromised. There was already an existing shortage of oxygen, and countries expected serious shortages of ventilators, medications, and workforce. The number of ventilators countries in Africa have ranges between 0 in some countries to close to 550 in some countries [10]. And based on these numbers, one can see that some countries have more ministers, vice presidents and officials than ventilators. And it is questionable whether these ventilators were working or not, if they were already occupied by other patients – trauma patients, stroke patients, etc.

Dr. Bekele described the fear felt by the surgical workforce, because receiving patients in this surgical setting is really dangerous. All surgical patients were not being tested before surgical procedures. There was a very serious shortage of PPEs, and the shortage was very serious. Anesthesia machines, which can be repurposed to serve as ventilators, did not have viral filters. With regards to operating theaters, most of the theaters are just large rooms converted to theaters, so negative pressure is more or less a luxury. And a shortage of N95 masks was a real issue. Providers could not begin to think about sterilization, reusing of N95s, yet.

Dr. Bekele then highlighted how COVID had completely disrupted undergraduate and medical education in the region. Only in a few handful of universities, like the University of Global Health Equity, had converted teaching to e-learning. Internet is a real issue, so e-learning is more of a luxury than a norm. Residencies were suffering significantly because there were limited morning meetings, limited teaching sessions, and elective cases had been cancelled. And there was serious fear among trainees about the spread of the disease. The three surgical colleges in Africa – College of Surgeons of Eastern, Central, and Southern Africa (COSECSA), West African College of Surgeons, and the College of Medicine of South Africa – had all either canceled their exams for the whole year or postponed them significantly, or seriously modified the way exams were being administered.

Dr. Bekele closed by emphasizing how COVID had tested the preparedness for this pandemic and how it seemed that surgical and anesthesia care preparedness was an indirect indicator for COVID preparedness. He lamented that global surgery voices have been silenced, understandably, for the past few months because of the immediate need to address is now the pandemic. Strengthening SAO care and preparedness in the continent is mandatory, not only to address surgical and obstetric problems, but to address issues like pandemics, like COVID. For the past 6 years, Africa has been engaged in the development national surgical anesthesia plans but did not take pandemics like COVID seriously in the process, and Dr. Bekele emphasized that this probably should be considered very seriously.

### Dr. Paul Farmer’s reflections

The the numbers that Dr. Bekele started with are impressive. Though it is likely not complete information, 100,000 cases are not anywhere near as many as we feared at this point, and nor are 3000 deaths. This case fatality rate would compare unfavorably to South Korea, Taiwan, or Germany, perhaps, but it would compare very favorably to the United States, where some states reported a case fatality rate over 5%. So, we still have that mystery – is this because of limited testing? Is there a milder form of the disease? Is this before an imminent spike? It has also been mentioned that the difference could be due to the differing age structures in Africa as compared to, for example, Italy and the United States.

Disturbing reports from Kano, Nigeria serve as a reminder that one of the ways to look at how much testing is being done is the fraction of cases that come back positive. Ideally, when testing is ramped up, it is going to be a fraction of the patients who are testing positive for the virus. Going back to Ebola, or HIV, early in those epidemics (one a pandemic, one an epidemic) – when tests started to be used in a rural area in a clinical setting, the majority of tests were often positive because they were done on people who were sick and looked like they had the disease. But once there were larger-scale studies, a very different picture emerged, and the fraction of positive cases decreased. In Kano, based on some reports, the majority of tests done were positive. That is the way it was in the early stages of the Ebola epidemic as well.

As Dr. Bekele stated, the word “elective” as in surgery does not mean frivolous, unnecessary, unimportant. It really means, in PGSSC circles, that very important and life-saving procedures are not happening. Another thing that has been brought out is the challenges inside a country once there has been a lockdown because of transportation difficulties. And again, this creates a new challenge to people who are seeking surgical or obstetric care, and it’s occurring on a continent, perhaps the only continent, where more than half of people still live in rural areas. That number is going down very rapidly, but it is probably still the most rural continent. These delays caused by interrupted transport are going to be devastating. It is an important reminder that people are very worried, and there is no reason not to be worried regarding fear of their own exposure. We hope that this fear will not be translated to decreased surgical access and hope even more that it will not be translated into increased risk for care providers.

We should be using some of our surgical preparedness metrics as a way of marking preparedness for the pandemic. The expression “canary in the coal mine” applies here – meaning these could be sensitive indicators, more sensitive than the blunt instruments we sometimes have. It is mandatory that we make a number of these changes and get patients back on track for their surgical care.

Previous discussions about Ebola and surgery are applicable here, in part because the second meeting of the Lancet Comission was in the medical desert in Sierra Leone. The first time that I set foot in Sierra Leone was in the company of surgeons in June 2014, and at that time, there was not universal enthusiasm among those present for the Lancet Commission to use infectious pathogens as a reminder of the importance of surgical care. But I hear that objection softening today.

### Two months later

As of July 29,2020, the number of cases of COVID in Africa had risen sharply to 871,970 and 18,475 patients have died [7]. In some countries, community spread was close to out of control and hospitals and ICU services are almost full. With regards to access to elective and emergency surgery, little had changed. As countries struggled to open their economies and borders, and as the number of cases continued to increase sharply, surgical services were still struggling to re-open to elective cases. Most elective services remained cancelled.

Despite these guidelines, hospitals in the region rarely tested patients for COVID due to the limited testing service. PPEs and other resources were still in short supply, and there were even reports of spread of COVID among cluster of health care providers in some countries. Fear among the surgical workforce remains a very real thing. There were reports of clustered outbreaks among health care providers in some countries, and Dr. Peter Matthew, the first surgical trainee in Sierra Leone, died due to COVID a few weeks prior. Shortage of PPEs and testing was still a major issue in most countries.

Finally, very little had changed with regards to the effect of COVID on undergraduate and medical education. Undergraduate education was still shut down in most countries with unclear reopening plans. Surgical residencies were significantly compromised, and college exams were converted to online clinical examinations at COSECSA. Fellowship level exams were also cancelled. Universities like the University of Global Health Equity (UGHE) were managing to continue their training through e-learning.

## Surgical systems strengthening in the COVID era: the way forward – Kee B. Park, MD MPH

Up until now, it has been argued that sufficient treatment capacity during pandemics saves lives, and the goal behind the “flatten the curve” is really to keep the peak demand for medical care from exceeding current health system capacity. But Dr. Park proposed that at the same time, it makes sense to find ways to *increase* supply of medical care, and we have reviewed here some of the strategies that are being used. The world has learned that the existing surgical capacity, with a space (such as the ORs, pre- and post-op units, ICUs and surgical wards), stuff (such as PPEs, ventilators, and sterilization equipment), and staff (such as anesthesiologists, whose services are in high demand this time, nurses, and surgeons), represent some of the most valuable assets in being able to rapidly convert into treatment capacity.

If you imagine a health care system as a piece of luggage, the surgical subsystem is like the expandable zipper that, when you open it, gives immediate increase in capacity -according to a report from McKinsey, perhaps as much as a 30% increase [11]. So Dr. Park asked whether, as global health practitioners, we can frame surgical capacity-building in low- and middle-income countries (LMICs) as a valuable component of pandemic readiness strategy. The PGSSC not only thinks so, but we argue that investing in surgical capacity building is one of the best buys for pandemic preparedness plans.

So why is this new framing important right now? It is because the amount of donor funding that’s going into COVID-19 response is unprecedented. According to the Kaiser Family Foundation, as of April, donors – including governments, development partners, and private foundations – have committed almost $20 billion for assisting with international COVID-19 response [12]. It is important to remember that this amount is only the assistance in health sector, and does not include the massive sums earmarked for economic stimulus, which is in the trillions [13]. The US $2 trillion dollar stimulus bill which was passed actually includes $157 billion directly allocated to health systems and research [14]. This means that the global development assistance for health, which has been hovering around the $30 billion a year range, will dramatically double or even triple post-COVID.

Knowing this, Dr. Park aimed to lay out a potential roadmap – not the only way, but one way we can do this. To convince pandemic donors to invest in surgical capacity-building in LMICs, Dr. Park emphasized that we must speak in a language that they will easily understand.

Those outside of the global surgery community may not easily grasp the current indicators that we use for surgical care. Decision-makers within the pandemic readiness community may not easily grasp concepts such as two-hour access, SAO (surgeon, anesthesia, obstetrician) density, and even volume of surgery as measures of surgical capacity. What they do understand is bed counts and number of ventilators. In fact, these are the metrics used by the Institute for Health Metrics and Evaluation when they started their model and then put it up on their website to predict the amount of treatment capacity at the start of the COVID- 19 outbreak. They explicitly look at ventilators and beds.

Taking this into account, Dr. Park along with some of his collaborators converted the unmet gaps in achieving the Lancet Commission target of 5000 procedures per 100,000 people into the number of additional surgical beds that are needed for each country in the LMICs. For example, they estimated that Afghanistan will need between 6000 to 10,000 additional surgical beds to meet the inpatient demands for the target of 5000 per 100,000 people. They are now working on estimating the additional OR and ICU needs, as well as the stuff and staff requirements for a functional surgical bed unit. This will provide us with a burden measure for each country and could feed simply into the allocation formula, similar to what Global Fund uses for their HIV, TB, and malaria funding. They are also working out the cost estimates for a fully functional and staffed bed so that the funding gaps for each country can be calculated.

Some may say this sounds like a global fund for surgery, and Dr. Park agreed. He recognized that there are valid reasons to be wary of yet another global fund for something, but surgical care, with its unique role within the health system and the potential for new resources to be mobilized, could significantly benefit from a pooled fund that oversees sustainable surgical capacity building processes in LMICs. In fact, we already have a global fund for surgery, it is called the Global Surgery Foundation (GSF), and it is housed within the United Nations Institute for Training and Research (UNITAR). Geoff Ibbotson, a trauma surgeon and the executive director of the GSF, led the launch of this Global Surgery Foundation in January 2020 at Davos at the World Economic Forum.

PGSSC, as a World Health Organization collaborating center for surgical system strengthening, has been involved with development of national surgical, obstetric, and anesthesia plans, and as Dr. Bekele mentioned previously, it is important at this point, post-COVID, to embed pandemic readiness strategy within these national surgical plans. If the Global Surgery Foundation is successful in raising enough money, they can facilitate the building of surgical capacity in LMICs that leads to overall health systems strengthening as well. Going back to the analogy of the piece of luggage – the whole luggage needs to be built up, and the expandable zipper, the surgical system, is part of that. Surgical system strengthening, done in an integrated way, not only improves pandemic readiness, but also, and, just as importantly, promotes equity in surgical care delivery.

### Dr. Paul Farmer’s reflections

I hope that this audience is going to push for this new framing of surgical capacity building as one of the best investments for pandemic preparedness. Even if it does not stick, even if it does not galvanize those controlling the purse-strings, it is an important exercise, nonetheless. And I appreciate the suggestion of going to where the money is. We do need to do this, because the alternative, of course, is to continue with our usual mechanisms of financing surgical care, which are out-of-pocket expenditures, impoverishing for families and the primary barrier for people who need surgical care and never get it.

## Panel discussion

### Approaches to safe and ethical reintroducing surgical services during a pandemic

#### Dr. Abebe Bekele, Rwanda

As of May 2020, Rwanda was still dealing with the initial surge and the peak was not yet reached. It was expected to be in around 3 weeks. But in the 6 days prior, numbers were steadily increasing—they were tripling, quadrupling. And countries like Rwanda had controlled it significantly. In fact, zero cases were being reported despite very vigorous contact tracing, testing. But the region in general, countries such as the Sudan, Ethiopia, Djibouti, and Kenya had seen sharp increases in the number of cases. So at the time of the webinar, they are in damage control mode.

Unfortunately, the mandatory preconditions were still a serious issue there. Ethiopia was doing close to 4000 tests a day - what the country needs is close to 50,000 a day. So even suspected cases were likely not getting tested at that moment, let alone surgical patients being tested before any surgery. PPEs were not available right and left, so professionals were very worried – would they be contaminated? The equipment they used, the ventilators, the theaters were not prepared to handle such issues. So Dr. Bekele proposed that instead we should talk about how to prepare to resume services than define what “electives” and “emergencies” are. The past few months were a learning curve. Now, he argued, they understood better what they were dealing with and how to prepare.

#### Dr. Nivaldo Alonso, Brazil

In private hospitals in Brazil, elective procedures were technically allowed to be performed at the time of the webinar. There were ORs are available for all types of cases. The problem is that they were asking for a COVID test 48 h before, which means that in reality, providers could not schedule just any case. Additionally, patients did not want to undergo surgery, and surgeons were afraid of legal issues that may arise if patients develop surgical complications during this time. Another concern was availability of ICU beds during this time. If a patient underwent an elective procedures in a private hospital, and then they required specialized or intensive care, where the risk of contamination was unclear. Given these concerns, it seemed preferable to avoid doing any of these procedures until the pandemic wa more under control.

Dr. Alonso stated that Brazil was dealing with some very difficult issues right now, including differing opinions of political leaders on how to handle the pandemic. While the Brazilian president had pushed for resumption of normal activities, governors and local councils were asking for a more cautious approach. It led to a very paradoxical situation in Brazil health care – private hospitals were asking surgeons to resume normal activities, while public hospitals are diverting all resources to COVID.

#### Dr. Alex Peters & Dr. Alaska Pendleton, United States

In New York, Dr. Peters described that hospitals had started reopening and doing cases, and they did in fact continue to do cases through the surge – Cornell kept five ORs open for emergent cases and were doing cases throughout the entire crisis. But there were very strict criteria for which cases could move forward. As the COVID census decreases, there may be an opportunity to reintroduce some of these surgical services. They were beginning to ask how could they start to fill the beds again with the needs that are in the community.

Discussions around reintroducing surgical services were centered around a few different things. The first was really all just a question of resources, which includes factors such as the capacity of intensive care units (ICUs). For example, cardiac surgery patients who were having symptomatic cardiac disease usually needed ICU care postoperatively, and if ICU space was not available, they could not restart cardiac cases. And other specialties such as vascular surgery would be subject to similar concerns. All of this was very tied into reopening of cities as well - for instance, when cities were closed, the number of trauma cases dropped significantly. As the city reopened and people went out more, especially as the weather improved, Dr. Peters and his colleagues saw upticks in trauma, and trauma is hugely resource-intensive. Additionally, there were a number of conditions that can be managed both operatively and nonoperatively, and appendicitis is really the best example of this. There has been a long-term debate about appendicitis, about whether the appendix should be taken out or it should be treated medically, as is done in much of Europe [15]. The question turns to what uses the lowest number of hospital resources, putting someone on antibiotics and trying to send them home fairly quickly, or just moving forward with an appendectomy?

There have been a number articles in the New York area about the drop in the number of patients presenting with acute coronary syndromes [16]. There was a concern that people were afraid to come to hospitals and were actually dying in their homes. But anecdotally, Dr. Peters and his colleagues saw drops in things like appendicitis, and it is difficult to explain how the incidence of something like appendicitis would decrease.

Then there was a question of can we start doing elective, urgent, or emergent ambulatory cases, like breast cancer surgeries that are often done in the ambulatory setting. If we do not have the inpatient beds, maybe that is an option. But should these cases be prioritized over those that require inpatient stays? So all those questions had come into play, and there has been no sure answer. Each hospital in New York has been tasked with doing this figuring that priority out on its own within the government’s mandates. But the hospitals were slowly reopening and trying to do it in a very thoughtful way.

In Boston, Dr. Pendleton described that her institution had to open a few more ICU beds and a few more ORs than New York City, given the different burden. So they had still been doing some of what could be called “elective” cases throughout, such as breast cancer and colon cancer cases, though in very limited number. At one point the number of active ORs was down to seven. As the city was reopening, patients were being referred to a city north of Boston, to Danvers, where they are opening for elective cases which would not require an ICU, and where they would have minimal exposure to this larger health care system.

#### Dr. Vatshalan Santhirapala, United Kingdom

The NHS had said that elective cases can restart on the 1st of June, which meant a break of 3 months. Dr. Santhirapala and his team had been analyzing the risk on several levels. On the national level, in the UK, the government essentially bought out all the private hospitals where elective surgeries could continue. Patients at these hospitals were required to have a COVID swab 7 days pre-op, self-isolated for 7 days, had a COVID swab on admission, and then were operated on the next day. So a very stringent protocol was introduced, which allowed all of the elective operating to continue in a clean pathway.

At the regional level, hospitals and providers were constantly assessing the current status of the pandemic in their region. If the pandemic is exponentially increasing, it is likely a better option to delay elective surgeries to preserve the capacity of the regional health system. On an institutional level, the main priority is providing a clean pathway like the one described above. Elective surgery patients must be separated from COVID patients in a safe manner so that risk of nosocomial COVID is avoided.

On the individual level, there is a risk to the individual of perioperative complications from COVID. The COVIDSurg collaboration has reported a 19% perioperative mortality in those who have COVID, an extremely high perioperative mortality in the COVID burden [17]. Secondly, there was the risk of deferring surgery. Those who have conditions such as cancer need to be risk- stratified. All of these risks, from the national to the individual levels, need to be understood to make those decisions.

### Surgical care delivery and social equity in the COVID era

#### Dr. Vatshalan Santhirapala, United Kingdom

In terms of social inequity, Dr. Santhirapala expressed his surprise at what had been seen in the UK. Despite having a fully “socialist” health care system where anyone can access health care whenever they want, they actually have seen that the black, Asian, or minority ethnic (BAME) population were younger than the Caucasian population that were dying, despite the fact that older age is one of the strongest risk factors for mortality. Despite being younger, the BAME patient was also dying.

The role of socio-economic deprivation cannot be ignored. Having a lower income and lower educational status means less access to knowledge of health care and health maintenance behaviors like good nutrition. This in turn manifests itself in being obese, or having hypertension, or having cardiac disease. We still don’t know this disease, and we don’t know its underlying pathways, but, as previously mentioned, the role of socioeconomic disparity in this disease process cannot be ignored.

#### Dr. Alex Peters & Dr. Alaska Pendleton, United States

It is important to recognize that the group of patients who are presenting with COVID are very different than the overall population. Being able to socially distance is a privilege, a privilege of individuals who have money, who have stable jobs, and who have stable housing. So it is no surprise that we saw that the hardest hit areas are those of greater social inequity.

This has also brought into very high relief the social inequities that do exist in New York. Many residents of Manhattan, who tend to be of higher socioeconomic status, had left the city, while those in Brooklyn, Queens, and the Bronx, faced a very different situation. And at the time of the webinar, providers were only beginning to understand these disparities and the role they have played in the pandemic.

New York has four different types of hospitals in the city – specialty cancer and orthopedic centers, academic medical centers, public hospitals, and private community hospitals. And the case fatality rate in each of those different types of hospitals, both based on the type of hospital and the populations each of those hospitals serves, varied dramatically [18]. There have been times when these varied the New York hospital systems have worked well together, but times in which they worked independently as well. And because of this, the idea that New York, as a city or as a region, might have a larger, better equipped health system with which to serve its population has not been realized. There had certainly been a lot of coordination by the Department of Health, but it made a difference which hospital patients wound up at and which hospital the ambulance brought them to during this pandemic, in terms of the chances of survival. It is something that needs to be looked at very closely and try to understand better as we move forward.

#### Dr. Paul Farmer’s reflections

Those on the frontlines have been alarmed, as we all have, by these high case fatality rates, but also by the fact that they vary so widely. And of course, this is what social medicine has focused on for the last 150 years-- who lives and who dies.

In West Africa, during the Ebola epidemic, the international public health community were clinical nihilists – that is, they argued against worrying about variations in case fatality rates among different populations, saying that all who get infected were likely to die. Just worry about stopping the spread of Ebola. It was the classic prevention versus care, as opposed to the integration of prevention and care. In Massachusetts, and in the United States in general, there was a wave of “prevention nihilism” or “containment nihilism.” People were saying, well, it’s too late now to do contact tracing. This cannot be the case, and the global health and global surgery community must go back again and again to the question of varying case fatality rates, too, and to combat ideas like clinical nihilism and containment nihilism. There will be many attempts to pass off these shocking disparities to either a culturalist explanation or to biological determinism. Those should be diagnoses of exclusion. We know that the social disparities are determining disparate outcomes, but there will be a rush to find some explanations that are either culturally deterministic or biologically deterministic. And the lead social medicine surgeons in the world should be prepared to discuss this very critically.

There another theme that arises throughout these talks - failing to be prepared. And yet there is so much information, and it comes through in the richness of these presentations. These health systems and clinical details are reminiscent of the discussions that we had around Ebola. Everything was “new”. If it was not a novel virus, it was certainly new to West Africa. But there is evidence to show that this was not the case. First of all, antibody studies suggest that Ebola had been in upper West Africa prior to the 2014 outbreak, which probably began in 2013. So, too, it is clinically. If you look at the so-called clinical surprises from the Ebola epidemic, you could also look back into the medical literature and find that these surprises had already been described in the literature. And so how can we make that claim when we have an entirely novel virus? Well, it is not the first – it is a SARS virus. And so when we hear about hypercoagulability, cardiac involvement, encephalopathic presentations, it is very likely that this has been described with related viruses, and finding out what’s similar and what’s different is an essential task. The goal of these conference proceedings is to highlight and to preserve these perspectives, so that they can be shared and used to better prepare both HIC and LMIC health systems for the future.

## Conclusion – a call to action

The expert perspectives shared during this seminar highlighted a number of key themes. From the frontlines came accounts of the need for increased critical care capacity, forced reallocation of hospital resources away from surgery leading to postponed or cancelled surgical cases, interruption of medical training, and obstruction of obstetric delivery care. There were also descriptions of innovated solutions to combat these new obstacles, such as repurposing of ORs to care for ICU patients, interdisciplinary teamwork to reduce staff exposure to COVID while improving patient outcomes, and strategic planning to be able to meet the sharp increases in patient volume across a hospital system. Then our speakers stepped back to look at SAO care and its role in pandemics on a broader level, not only in terms of health system strengthening and preparedness, but also highlighting the weakness and social inequities that were revealed.

A vaccine will be wonderful, but that will not be good enough. It won’t be good enough because of something Mark Twain said: “History never repeats itself, but it rhymes.” Even if we do have a vaccine, there will be something else in 6, or 12, or 18 months that is going to rhyme with coronavirus – maybe not to our ears, but to our systems. And so whether we’re talking about the equity issues and the social issues or the role of surgery in coronavirus, how do we think differently about our approaches at every level to such a crisis, whether it is Brazil, or the United States, or Rwanda. How do we structure our hospitals and healthcare systems differently, so that when the next crisis comes up, we are not spinning around in circles trying to reinvent what some of the presenters described today? How do we think differently about not just coronavirus, but the next obstacle that appears? How do we fund things differently so that we’re not asking for people for out-of-pocket payments when the next emergency arises?

This is a call to action to make sure that we actually act, not just to address this pandemic, but to prepare health systems for the future, to change the role of surgery in pandemic preparedness and health security, and to eliminate the social inequities laid painfully bare in the height of COVID.

## Data Availability

NA – a recording of this meeting is available online at https://www.pgssc.org/covid19surgerywebinar.

## Abbreviations

BAME: Black, Asian, or minority ethnic
COBRA team: COVID-19 Bundled Response for Access team
COSECSA: College of Surgeons of East, Central, and Southern Africa
GSF: Global Surgery Foundation
HIC: High-income country
IPC: Infection prevention & control
ICU: Intensive care unit
LMIC: Low- and middle-income country
MGH: Massachusetts General Hospital
NSOAP: National surgical, obstetric, and anesthesia plan
OR: Operating room
PGSSC: Program in Global Surgery and Social Change
PPE: Personal protective equipment
SAO: Surgery, obstetrics, and anesthesia
UGHE: University of Global Health Equity
UNITAR: United Nations Institute for Training and Research
